# Response to “Upping the dose of dementia risk reduction”

**DOI:** 10.1002/alz.12693

**Published:** 2022-05-16

**Authors:** Sylvie Belleville, Simon Cloutier, Samira Mellah, Bruno Vellas, Sandrine Andrieu, Nicola Coley, Tiia Ngandu

**Affiliations:** ^1^ Research Center, Institut Universitaire de Gériatrie de Montréal Montreal Canada; ^2^ Psychology Department, Faculty of Arts and Science, Université de Montréal Montreal Canada; ^3^ UMR1027 Inserm, University of Toulouse III Toulouse France; ^4^ CHU de Toulouse Toulouse France; ^5^ Center for Epidemiology and Research in Population health (CERPOP), University of Toulouse Toulouse France; ^6^ INSERM UMR1295, UPS Toulouse France; ^7^ Department of Epidemiology and Public Health, Toulouse University Hospital Toulouse France; ^8^ Department of Public Health and Welfare, Finnish Institute for Health and Welfare (THL) Helsinki Finland

1

Timothy Daly commented on our article reporting a dose effect in a multidomain intervention to prevent cognitive decline in older adults at risk of dementia.^1^ Our article modeled the dose‐response relationship in a multidomain intervention and identified an optimal dosage and dose differences based on individual characteristics, such as level of frailty and education. Although Daly recognizes that the approach is of value, he questions whether efforts would be better invested to address the social determinants of brain aging.

Dementia is a complex disease the cause of which is probably multi‐determined, with contributions from both the detrimental impacts of neuropathology and neuroprotective factors. It appears that personal and environmental characteristics contribute to its expression. For this reason, understanding the conditions that promote a healthy brain will require that different fields, including philosophy and sociology, put cognitive health on their agenda. We see this commentary as an important part of the interdisciplinary dialogue that should take place around these issues.

We strongly agree that brain health and dementia prevention need to be part of the public health agenda and that broader social approaches must be implemented to promote brain health including actions to address the social determinants of brain health. Evidence shows that living in a low‐income neighborhood is associated with an increased risk of dementia, and certain living conditions (e.g., access to stores with healthy foods and walkability) have been identified as barriers/facilitators to the adoption of a lifestyle that is beneficial for the brain.

However, we do not see the social and personal approaches to brain health as being in opposition. For instance, longer formal time in school is associated with better cognition later in life but some studies found that it is the case for men but not for women,^2^ illustrating the interaction between social and individual characteristics. Furthermore, individuals are driven by social and psychological factors, and are key agents of their health. Personal factors such as self‐efficacy, empowerment, and education are critical dimensions and have been shown to determine individual health. Public health organizations are aware of this and contribute to awareness campaigns, as individuals’ adherence to public health messaging is essential to induce personal and societal changes.

The types of multi‐domain interventions that we are proposing may be one of the tools that society can provide to older adults. Here, an analogy can be drawn with formal education programs. Today, no one would question the importance of providing children with an educational program that equips them at the cognitive level. Thus one would think that providing late‐life education might be as critical for the aging brain as for the developing brain. In addition, by modifying cognition through lifestyle improvements, these interventions contribute to dementia literacy, self‐efficacy, and empowerment. They can be offered at all levels in the community, and studies are initiated with a participatory approach to assess how they can be implemented and/or adapted to meet the needs of older adults and community organizations. But of course, for these programs to be implemented, their effectiveness must be demonstrated through sound design.

Timothy Daly, mentions that looking at dose effects characterizes a “pharmacological” model rather than more socially oriented models. However, dose models are not irrelevant in public health approaches. There are many examples where dose is incorporated into public health recommendations, such as for alcohol consumption, toxicants, or sun exposure. But this is also true for more general or social conditions: For example, many countries have introduced compulsory schooling up to a certain age with the idea that there is a minimum dose of education that should be beneficial to the individual. All these recommendations stem from the fact that dose is an important parameter, not only in a biological context but also for more complex socially determined conditions.

In summary, we agree that effective dementia prevention at the population level will require a public health approach, and that randomized controlled trials are much more challenging to perform in the context of long‐term lifestyle interventions than for a standard drug versus placebo trials, but this does not mean that such trials are not helpful or indeed necessary. Investigating individual factors does not prevent consideration of social factors. In our view, these factors are inherently interdependent and mutually reinforcing. It is essential that researchers from different perspectives and disciplines continue their conversation about dementia prevention.

## CONFLICT OF INTEREST

SB is a consultant on dementia prevention for Lucilab Inc. BV is investigator and consultant for Roche, Biogen, Lilly, Esai, and taurx, outside the scope of the present paper.
